# The effect of methylphenidate-OROS^®^ on the narrative ability of children with attention-deficit hyperactivity disorder

**DOI:** 10.4102/sajcd.v64i1.180

**Published:** 2017-02-27

**Authors:** Tessa L. Rausch, Diane L. Kendall, Sara T. Kover, Elizabeth M. Louw, Ursula L. Zsilavecz, Anita van der Merwe

**Affiliations:** 1 Department of Speech-Language Pathology and Audiology, University of Pretoria, South Africa; 2 Department of Speech and Hearing Sciences, University of Washington, United States; 3 Department of Statistics, University of Pretoria, South Africa

## Abstract

**Background and objective:**

Children with attention-deficit hyperactivity disorder (ADHD) experience difficulty with expressive language, including form (e.g. grammatical construction) and content (e.g. coherence). The current study aimed to investigate the effect of methylphenidate-Osmotic Release Oral System^®^ (MPH-OROS^®^) on the narrative ability of children with ADHD and language impairment, through the analysis of microstructure and macrostructure narrative elements.

**Method:**

In a single group off–on medication test design, narratives were obtained from 12 children with ADHD, aged 7–13 years, using wordless picture books. For microstructure, number of words, type–token ratio and mean length of utterance were derived from narrative samples using Systematic Analysis of Language Transcripts conventions. For macrostructure, the narratives were coded according to the Narrative Scoring Scheme, which includes seven narrative characteristics, as well as a composite score reflecting the child’s overall narrative ability.

**Results:**

The administration of MPH-OROS^®^ resulted in a significant difference in certain aspects of language macrostructure: cohesion and overall narrative ability. Little effect was noted in microstructure elements.

**Conclusion:**

We observed a positive effect of stimulant medication on the macrostructure, but not on the microstructure, of narrative production. Although stimulant medication improves attention and concentration, it does not improve all aspects of language abilities in children with ADHD. Language difficulties associated with ADHD related to language content and use may be more responsive to stimulant medication than language form, which is likely to be affected by cascading effects of inattention, hyperactivity and impulsivity beginning very early in life and to progress over a more protracted period. Therefore, a combination of treatments is advocated to ensure that children with ADHD are successful in reaching their full potential.

## Introduction

Attention-deficit hyperactivity disorder (ADHD) is a frequently occurring psychiatric condition in school-aged children with estimates of prevalence ranging from 3% to 5% (National Institutes of Health [NIH] Consensus Development Panel, 2000; Westby & Watson, 2004). As outlined in the Diagnostic and Statistical Manual of Mental Disorders, 5th edition (DSM-5; American Psychiatric Association, 2013), ADHD refers to a heterogeneous group of individuals who display a persistent pattern of inattention, with or without hyperactivity and impulsivity, that disrupts functioning or development. Based on the presentation of these difficulties, individuals may be considered to belong to one of three subtypes: ADHD primarily inattentive (ADHD-PI), ADHD primarily hyperactive or impulsive (ADHD-PH) or ADHD combined type (ADHD-C). The symptoms of ADHD are pervasive and negatively impact performance across a variety of settings throughout the individual’s life. Although widespread agreement exists regarding the validity of ADHD as a diagnosis, there is not a single neurological or physiological test to objectively diagnose the disorder. Furthermore, no definite neurological, genetic or biological aetiology exists (Furman, 2005; NIH Consensus Development Panel, 2000).

Theories regarding the aetiology of ADHD implicate neuroanatomical, neurochemical and neurophysiological mechanisms. In particular, dopaminergic neurotransmitters, for example, catecholamines (i.e. hormones produced by the adrenal glands which include dopamine, norepinephrine and epinephrine; Dugdale, 2013), have been shown to regulate cognitive behaviours such as attention, inhibition and motivation (Ballard et al., 1997). Though the exact effect of the catecholamines on behaviour remains unresolved, support for their involvement can be found by improved performance of children with ADHD across a range of behavioural measures and cognitive tasks, including those of attention and memory, following stimulant medication (Berridge & Waterhouse, 2003; Pelham et al., 1990a; Schachar et al., 2008).

In addition to impaired attention and inhibition, children with ADHD frequently present with speech or language difficulties. Although most estimates range between 20% and 60% (Oram, Fine, Okamoto & Tannock, 1999), reports on the rate of speech or language impairment in children with ADHD vary depending on whether the samples were clinically referred or enlisted from the community (Engelhardt, Ferreira & Nigg, 2011). Children with ADHD demonstrate impairments in language processes that have been noted in verbal production (Oram et al., 1999; Purvis & Tannock, 1997), comprehension (McInnes, Humphries, Hogg-Johnson & Tannock, 2003) and reading (Baker & Cantwell, 1992). Prior work documenting disorders in language processing in this population has focused almost entirely on the modality of expressive language, documenting impaired sentence formulation and organisation, coherence and self-monitoring (Francis, Fine & Tannock, 2001; Purvis & Tannock, 1997), poor topic maintenance (Tannock, 2004/2005; Westby & Watson, 2004) and increased grammatical errors and dysfluency because of false starts, repetitions and hesitations (Tannock, 2004/2005). Furthermore, children with ADHD are prone to speak for longer stretches (excessive talk) with many short pauses during speech production (Breznitz, 2003). These characteristics are likely because of verbal retrieval problems resulting in increased use of non-specific terms (Tannock, 2004/2005). Taken together, children with ADHD are at risk of having expressive language abilities that are characterised by weaknesses in both form (e.g. grammatical construction) and content (e.g. coherence).

The weaknesses in expressive language associated with ADHD are particularly apparent in the context of narrative production. For example, the narrative production of children with ADHD is characterised by errors in the sequencing of story events, which research has attributed to a breakdown in the global organisation of language (Purvis & Tannock, 1997). The literature also reports an inability to acknowledge the needs of the communication partner and a failure to achieve and monitor cohesion at a sentence level (Purvis & Tannock, 1997). Tannock (2000) discusses the fact that poor implementation of pragmatic rules negatively impacts this population’s spoken language, with many studies reporting difficulty in the application of basic pragmatic rules essential for successful and cohesive narrative production, including turn taking, introduction and topic maintenance (Oram et al., 1999; Purvis & Tannock, 1997; Väisänen, Loukusa, Moilanen & Yliherva, 2014). Furthermore, children with ADHD are prone to producing ambiguous statements during narrative production because of the unclear use of referents and a lack of cohesive devices, as well as providing far less information during narration than their normally developing peers (Miniscalco, Hagberg, Kadesjö, Westerlund & Gillberg, 2007; Purvis & Tannock, 1997; Rumpf, Kamp-Becker, Becker & Kauschke, 2012). In addition, van Lambalgen, van Kruistum and Parigger (2008) reported that children with ADHD use complexity-reducing strategies during narrative production. These strategies include more tenseless utterances by avoiding direct speech, a greater number of tense shifts leading to incoherence and a limited use of temporal adverbials. According to Luo and Timler (2008), children with ADHD and language impairment produce less organised narratives than their typically developing peers, with particular difficulty noted with story units, with Goal–Attempt–Outcome structure. Although Luo and Timler found no significant difference between the narratives produced by children with ADHD and their typically developing peers during a picture sequence task, they found that the children with ADHD and language impairment produced significantly fewer complete Goal–Attempt–Outcome units than their peers when narrating a single picture. As such, narrative tasks may most effectively illustrate the language difficulties, as well as the potential for improved performance because of treatment, in children with ADHD.

### Theoretical accounts for the association between ADHD and language impairment

The co-occurrence of attention disorders and language impairment is not arbitrary; however, there continues to be dispute regarding the specific cognitive-linguistic mechanisms responsible for language impairments in this population. Theories ranging from general developmental delays to executive function impairments to attention deficits have been proposed.

One explanation for the documented concomitance between these deficits is that both are rooted in general developmental delays, as indicated by studies that have focused on the relationship between the development of attention, cognition and language (Redmond, 2004). For example, Tallal, Dukette and Curtiss (1989) found high correlations between language, attention and motor functioning, suggesting that attention deficits identified in children with language disorders may be related to neurodevelopmental delays in perceptual and motor functioning. Boucher (2000, p. 13) suggested that developmental disorders associated with language difficulties, such as ADHD, may reflect a disruption in the development of underlying ‘time parsing mechanisms’ (referring to a continuum of perceptual and cognitive processes implicated in the segmentation and analysis of information, including linguistic material). The continuum of perceptual and cognitive processes described by Boucher (2000) could allow for the variation observed in attentional, cognitive and linguistic symptoms associated with ADHD.

Alternatively, the notion that language acquisition may be hampered by existing deficits in attention has been proposed (Camarata & Gibson, 1999; Sameroff & Chandler, 1975). Camarata and Gibson (1999) discussed the effects of ADHD on language acquisition through the transactional model of mother–child interaction, which focuses on the interaction between child and adult behaviour responsible for the development of a child’s language (Sameroff & Chandler, 1975; Yoder & Warren, 1993). Based on this model, the authors suggest that inattention, hyperactivity and impulsivity negatively influence a child’s ability to engage in language-learning opportunities, upsetting these interactions and therefore disrupting the process of language-learning. Although these disruptions occur early in life, they presumably continue through childhood, with cascading effects on more advanced language forms and uses.

In addition to general developmental delays and attention deficits, executive dysfunction, which refers to those cognitive, self-regulatory behaviours necessary for the selection and maintenance of actions, guiding one’s behaviour within a rule-governed context, is also observed in this population (Barkley, 1997; Westby & Watson, 2004). Some propose that deficits in executive function are responsible for core behavioural symptoms of ADHD as well as language difficulties (Tannock & Schachar, 1996) and diminished working memory (Barkley, 1997). Furthermore, Tannock and Schachar (1996) suggested that this executive dysfunction may create a profile of language difficulties unique to children with ADHD. Support for this theory can be found in the work of Tannock (2004/2005) and Westby and Watson (2004), which showed that language characteristics of children with ADHD include a lack in ability to initiate or plan an intended message. This results in difficulty shifting between, and organisation of, their thoughts, while maintaining the necessary sequence of behaviours or events. The presence of these deficits could contribute to weaknesses in narration in children with ADHD, as these are the skills required to generate a rich and cohesive narrative (Moonsamy, Jordaan & Greenop, 2009).

### Narrative production in children with ADHD

Given the symptoms of ADHD and the underlying mechanisms thereof, the cognitive profiles associated with ADHD can be mapped onto the requisite skills of narration, which include sustained attention and topic maintenance, as well as complex syntax and an organisational structure based on temporal and causal chains (Owens, 2001). Given this overlap, it is evident that narrative production, in particular, would be informative to study with respect to the effects of medication on children with ADHD. The importance of investigating narratives has been highlighted in the literature for a number of reasons, perhaps foremost, because of the close correlation between narrative performance and academic success in children with language impairment. Research has indicated that preschool children with poorly developed narrative abilities are at risk for later academic and language difficulties (Paul & Smith, 1993). In addition, narrative skills are fundamental to social communication. Oral narratives enable individuals to develop social relationships through the sharing of experiences, allowing one to engage emotionally with others (Coupland & Jaworski, 2003). Furthermore, because of the decontextualised nature of narratives, individuals are able to share events that are removed from the here-and-now (Peterson, Jesso & McCabe, 1999). That is, the core behavioural difficulties and language impairments associated with ADHD could impact narrative production ability, with implications for academic and social outcomes.

The idea that medication could positively influence language production in narratives in children with ADHD has been documented in only a few studies. One such study is that by Francis et al. (2001), in which 50 children with ADHD, aged 7–12 years, listened to an audiotaped story accompanied by a wordless picture book during a randomised, placebo-controlled crossover trial with both 10 mg and 20 mg doses of standard-release methylphenidate (MPH). MPH is a stimulant medication used in the treatment of the behavioural symptoms of ADHD through its effect on neurotransmitter levels within the brain (Ballard et al., 1997; Poulton, 2006). Participants were required to retell the story as well as answer comprehension questions. The narratives were analysed in terms of their story grammar (critical narrative elements), length of retell and errors produced. Results indicated that MPH increased the participants’ recognition of the character’s internal responses and attempts (i.e. aspects of narrative macrostructure) but showed no effect on retelling errors, story length or story comprehension. Based on the design of the study, however, it is evident that participants’ understanding of the narrative was influenced by their comprehension of the audiotaped story prior to their narrative retell. Indeed, the way in which a narrative task is elicited (e.g. visual vs. audio + visual) can impact performance (Gazella & Stockman, 2003; Schneider & Dube, 2005). As difficulties in comprehension of the presented story may have influenced the participants’ ability to subsequently retell the narrative, results obtained would be a reflection of the effect of MPH on the summation of receptive and expressive language abilities.

In a similar study to that of Francis et al. (2001), Derefinko, Bailey, Milich, Lorch and Riley (2009) investigated the effects of stimulant medication on the narrative production of 17 children with ADHD, aged 9–13 years, using an online story narration task. Online narration allows for the investigation of narratives and story processing, while decreasing demands on memory and receptive language abilities. In this case, narrative elicitation involved telling a story from a wordless picture book, without first listening to a recording of the story or previewing the picture book ahead of time. Derefinko et al. compared the narrative abilities on and off-medication for children with ADHD who were taking a variety of stimulant medications, rather than a single stimulant across all participants. Narratives were evaluated with a focus on goal-based attempts and outcomes (i.e. goal-based story events), using story grammar categories that included overall goal, subsequent subgoal, attempts and outcomes as well as resolution of the overall goal. Furthermore, within-clause, whole-clause and repetition errors were noted. For children with ADHD, results indicated that stimulant medication did not improve goal-based story production skills. Although children on medication included more clauses in their narratives (increased length of narratives), no other significant effects were evident. Thus, in contrast to Francis et al., Derefinko et al. identified an effect of medication on microstructure (i.e. on productivity in terms of narrative length) – but not macrostructure – of narrative production in children with ADHD. The discrepancy between the findings of these two studies may have been a result of methodological differences in elicitation of narratives or the particular outcome measures examined.

The purpose of the current study was to extend prior work on the effects of a single medication, namely MPH-Osmotic Release Oral System (MPH-OROS^®^), on language production processes in children with ADHD. In the current study, so as to circumvent the effect of comprehension skills on narrative production, an elicitation procedure was utilised that was less likely to result in performance that varies on the basis of memory or receptive language ability. Narrative elicitation did not involve listening to the story in advance, but rather previewing the wordless picture book prior to narrative production, allowing the processing of the entire story before planning and organising story components into a cohesive narrative. The current study sought to comprehensively measure microstructure and macrostructure elements of narration, employing a sensitive and specific scale of narrative production that rates the degree of development of story grammar elements rather than the mere presence or absence thereof (Narrative Scoring Scheme; NSS; Miller, Adriacchi & Nockerts, 2011). To that end, in a group of 12 children with a diagnosis of ADHD and developmental language impairment, the following two questions were addressed: (1) Is there an effect of MPH-OROS^®^ medication on *microstructure* language production elements during story narration, as measured by productivity, grammatical complexity and lexical diversity? (2) Is there an effect of MPH-OROS^®^ medication on *macrostructure* language production elements (introduction, character development, conflict resolution, mental state, referencing, cohesion and coherence) during story narration, as measured by NSS?

## Method

### Participants

In the context of a single group off–on medication test design, 12 first-language English-speaking children with ADHD (3 girls and 9 boys), aged 7 and 13 years (mean age of 11.23 with a standard deviation of 2.28) with average or above average intelligence (mean of 96.42 with a standard deviation of 10.05), were selected from a private remedial school. See Table 1 for a summary of participant characteristics. Only those children with ADHD were included in the study as the primary aim of the current study is to investigate the effects of MPH-OROS^®^ on a heterogeneous group of individuals with ADHD. Written consent was obtained from parents prior to commencement of the study, and verbal assent was obtained from the children prior to each data gathering session. A university-based ethics committee granted permission for the study to be performed.

**TABLE 1:** Demographics of study participants.

Participant characteristic	Sub-test	Mean	SD	Range		

				*N*	Minimum	Maximum
Age	-	11.23	2.28	6.6	7.3	13.9
IQ (Van Eeden, 1997)	-	96.42	10.05	32	82	114
Clinical Evaluation of Language Fundamentals (CELF) (Semel et al., 2003)	Formulated sentences	7.50	3.06	12	1	13
	Understanding spoken paragraphs	8.08	3.80	12	1	13
	Familiar sequences	7.91	2.60	10	1	11
Test of Auditory Processing Skills (TAPS) (Martin & Brownell, 2005)	Word discrimination	11.00	1.35	4	9	13
	Phonological blending	10.17	3.76	14	1	15
	Number memory forward	9.84	2.29	8	7	15
	Number memory reversed	8.17	2.98	10	11	1
	Word memory	10.50	3.15	14	5	19
	Sentence memory	9.25	2.67	8	5	13
	Auditory comprehension	8.58	2.50	9	5	14
	Auditory reasoning	8.75	1.66	5	6	11
Peabody Picture Vocabulary Test (PPVT) (Dunn & Dunn, 2007)	-	3.25	1.29	4	1	5

Note: Norms for the TACL are only available up to the age of 10 years and available scores for participants, aged 7–10 years, are therefore not reflected in the table. Similarly, scores for the CELF sub-test ‘Concepts and Following Directions’ have been omitted because of a lack of norms for participants above the age of 12 years. CELF, TAPS and PPVT scores are represented by standard scores. For standard scores, mean is 10.

### Inclusion

Only those children who had been diagnosed with ADHD by a qualified child neurologist or child psychiatrist, using the *Diagnostic and Statistical Manual of Mental Disorders-IV* (*DSM-IV*), were included in this study. In addition, participants held a current prescription for MPH-OROS^®^ for a minimum of 3 months prior to the commencement of the study with consent from parents to administer medication at school. Participants had a diagnosis of language impairment, as defined by at least one standard deviation below the mean for standard scores on any of the following test batteries: Clinical Evaluation of Language Fundamentals (CELF-4) (Semel et al., 2003), Test of Auditory Processing Skills (TAPS-3) (Martin & Brownell, 2005), Peabody Picture Vocabulary Test (PPVT-4) (Dunn & Dunn, 2007) and Test for Auditory Comprehension of Language (TACL) (Carrow-Woolfolk, 1998). See Table 1 for performance data on these tests. Finally, study participants were currently receiving intervention for language difficulties by a speech-language therapist, during the course of the school day.

**FIGURE 1:**
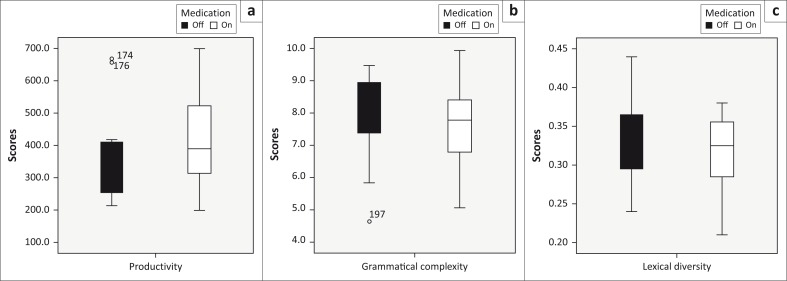
Boxplots representing the median and interquartile ranges for microstructure elements, off and on medication for (a) productivity (number of words), (b) grammatical complexity (mean length of utterance) and (c) lexical diversity (type–token ratio).

**FIGURE 2:**
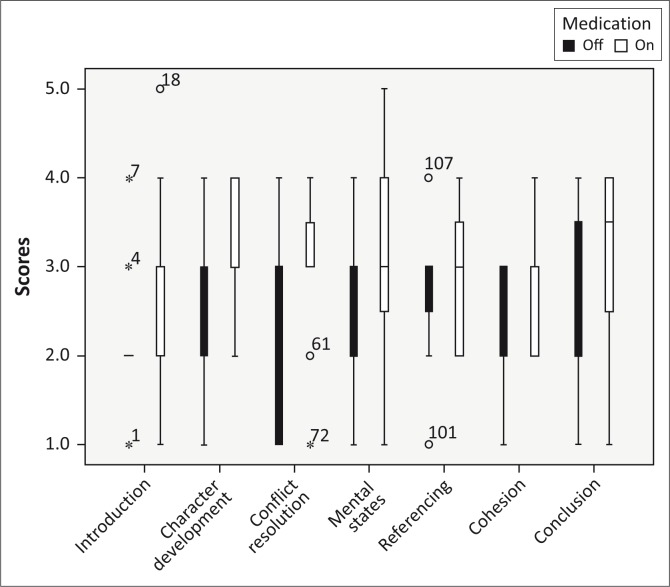
Boxplots indicating median and interquartile ranges for macrostructure elements, off and on medication.

### Exclusion

Individuals were excluded if they demonstrated below average intelligence based on the Senior South African Individual Scale – Revised (SSAIS-R) (Van Eeden, 1997) and also if they spoke English as a second language. Children receiving additional chronic medications were not permitted to take part in the study, as this may have clouded the results obtained.

### Procedure

Two examiners, the first author and one other speech-language therapist, carried out data collection in a quiet environment. To assess the behaviour without the influence of medication, all participants were assessed after a 2-day ‘drug holiday’. The assumption for this time period is that after 24 hours, normal blood level baselines would be reached (Liu, Muniz, Minami & Silva, 2005). Participants were assessed twice on a single day: once prior to receiving their daily dose of medication and again 4 hours later (after the medication had taken effect). The decision to schedule the post-medication assessment 4 hours after medication had been administered was made in consultation with a representative from the relevant pharmaceutical company, as this is when maximum level of MPH-OROS^®^ is reached.

### Narrative task and outcome measures

#### Narrative stimuli

The research questions were answered by the administration and subsequent scoring of language production elicited by narrative production procedures. The wordless picture books *Frog, Where Are You?* (Mayer, 1969) and *One Frog too Many* (Mayer & Mayer, 1975) were used to elicit narratives, based on evidence of their comparability (John, Lui & Tannock, 2003). Order of presentation of the books was randomised between pre- and post-medication sessions. *Frog Goes to Dinner* (Mayer, 1974) was used for warm up, prior to the pre-medication session, and the narrative obtained was recorded but discarded without analysis. These stories are similar with regard to theme, structural complexity, number of main characters and length (John et al., 2003; Petersen, Gillam & Gillam, 2008; Strong, 1998), and they have been used extensively to assess children’s narrative abilities (Berman & Slobin, 1994) with both typical and atypical populations (Losh & Capps, 2003).

#### Narrative production procedure

The book was placed on the table in front of the child. The instructions given to the participants were pre-formulated in order to avoid any additional influence on their performance:

Here is a picture book that tells a story. This book has no words. I want you to look through the book from start to finish. Then we will go through the book together and I want you to tell me the story for each picture.

If the child was quiet for prolonged periods of time, the prompt ‘Tell me more’ was used once. No further prompts to produce language were given. The examiner gave no feedback regarding performance but provided occasional social continuants such as head nods and ‘uh-huh’. Participant language samples, and all prompts from the examiner, were digitally recorded in a quiet environment using an Olympus VN-713PC Dictaphone to allow for later playback and analysis.

#### Transcription and outcome measure scoring

The first author orthographically transcribed the audio recordings into C-units using the Systematic Analysis of Language Transcripts (SALT) guidelines and software (Miller et al., 2011). For microstructure elements, the number of words, type–token ratio and mean length of utterance (MLU) in words, of complete and intelligible utterances, were determined using SALT standard measures. For the analysis of macrostructure, the transcripts were coded according to the NSS (Miller et al., 2011). The NSS includes instructions on how to code story grammar and cohesion according to a 0- to 5-point Likert-type scale. The NSS focuses on the following aspects of narratives, each described further below: introduction, character development, mental states, referencing, conflict resolution, cohesion and conclusion. Each category is assigned a score ranging from 5 (proficient), 3 (emerging) and 1 (minimal or immature). Scores of 2 and 4 were assigned if performance fell somewhere between the major anchors (Bajaj, 2007). A score of 0 is assigned when performance cannot be judged because of a variety of child errors including unintelligibility, task abandonment or refusal to complete the task at hand, conversing with the examiner and narration of the incorrect story (Miller et al., 2011). The scores from the seven categories were then combined to provide a composite score reflecting the child’s overall narrative ability as described by Miller et al.

**Outcome measure description:** Microstructure and macrostructure language elements were quantified. Microstructure was defined as productivity (number of words produced), lexical diversity (type–token ratio) and grammatical complexity (MLU). Macrostructure was defined using the NSS as introduction (provides the setting for the story and introduces main characters), character development (ability to use metalinguistic verbs, differentiate between characters and talk in first person to depict story characters), mental states (ability to use metacognitive verbs to describe thoughts and feelings), referencing (referential cohesion through the use of pronouns and antecedents), conflict resolution (highlights major conflicts and resolutions), cohesion (refers to lexical and conjunctive aspects which include ordering, emphasis and transition between story events) and conclusion (story is wrapped up using concluding statements) (Miller et al., 2011).

**Transcription agreement and NSS scoring reliability:** Intra- and inter-rater agreement and reliability procedures were performed by the first author and an unbiased speech-language pathologist, who was blinded as to whether recordings were pre- or post-medication and who had no personal interest in the study. These procedures were carried out on all microstructure and macrostructure elements on 20% of the data. Narratives from 5 of the 12 participants were randomly selected, from which one of the narratives (either pre-medication or post-medication) was randomly chosen for agreement and reliability scoring. Narratives were re-transcribed in SALT and re-analysed using NSS both by the original transcriber as well as by the second transcriber.

Inter(intra)-rater agreements for the SALT microstructure transcript elements were determined using Pearson’s correlations. The results showed the following correlation coefficients: 1.00 (1.00) for number of words, 0.997 (1.00) for length of *t*-unit and 0.987 (0.996) for type–token ratio.

Krippendorff’s alpha values were calculated with ordinal scaling to determine inter-rater reliability of the NSS scores using 0.67 (acceptable) and 0.80 (adequate) benchmarks (Hayes & Krippendorff, 2007). This reliability metric was chosen because it has been used extensively with NSS scoring (Finestack, Palmer & Abbeduto, 2012; Heilmann, Miller & Nockerts, 2010). The resultant alpha values for each NSS component are represented in Table 2.

**TABLE 2:** Krippendorff’s alpha values obtained for NSS scoring reliability.

NSS component	Alpha value
Introduction	0.25
Character development	0.99
Conflict resolution	0.64
Mental states	0.79
Referencing	0.79
Cohesion	1.00
Conclusion	1.00
Total macro elements	0.73

Note: Benchmarks: 0.67 (acceptable) and 0.80 (adequate).

## Results

Based on the small sample size and the skewed distribution of the differences between the on- and off-medication measurements, non-parametric tests were used. Inferential statistics were employed to determine the significance of results at a 5% level of significance. The Wilcoxon paired signed ranks test, a non-parametric equivalent of the paired *t*-test, was selected to determine the effectiveness of MPH-OROS^®^ on improving microstructure and macrostructure elements during narration. Multiple testing was done to evaluate the effect of medication on each of the independent microstructure and macrostructure language production elements separately. See Table 3 for descriptive and test results. The median (*me*) and interquartile range (*IQR*), the difference between the third quartile and the first quartile, have been reported because of the skewed distribution of the data. These descriptive measures are more robust than averages and standard deviations as they are not influenced by outliers.

**TABLE 3:** Effects of MPH-OROS^®^ on the narrative production of children with attention-deficit hyperactivity disorder.

Narrative components	Elements	Off-medication			On medication			Effect of medication		
		
		Median	Mean (average)	IQR	Median	Mean (average)	IQR	*Z*	*p*	*r*
Microstructure	Productivity (number of words)	301.5	361.25	166.5	389	410	216	−1.138	0.255	−0.232
	Lexical diversity (type–token ratio)	0.33	0.33	0.08	0.32	0.32	0.09	−0.747	0.455	−0.152
	Grammatical complexity (mean length of c-unit)	7.93	7.73	1.3	7.78	7.67	1.93	−0.118	0.906	−0.024
Macrostructure scored range of 5–1 (per Heilmann et al., 2010)	Introduction	2	2.17	0	2.58	2.58	1.0	−1.186	0.236	−0.242
	Character development	3	2.67	1.5	3.33	3.33	1.0	−1.522	0.121	−0.316
	Conflict resolution	2	2.17	2.0	3	3.00	0.8	−2.226	0.026	−0,454
	Mental states	2.5	2.5	1.0	2	3.25	1.8	−1.538	0.124	−0.314
	Referencing	3	2.75	0.8	3	2.92	1.8	−0.632	0.527	−0.129
	Cohesion	2	2.25	2.0	3	2.83	2.0	−2.333	0.020	−0.476
	Conclusion	2.5	2.58	1.8	3.5	3.16	1.8	−1.552	0.121	−0.317
	NSS total score	16.5	17.08	4.0	21	21.08	5.0	−2.673	0.008	−0.546

Note: *Z*-value represents results for the Wilcoxon signed ranks test for the difference between performance on and off-medication.

The NSS categories, in accordance with previous literature, were divided into three groups. Introduction, Conflict Resolution and Conclusion were grouped together as these NSS components refer to the key components of story grammar. Referencing and Cohesion form the second group and represent the overall coherence of the narrative. Character Development and Mental States are concerned with the use of a literate style (Rollins, 2014). We used the Holm (1979) sequentially rejective multiple comparisons procedure for each of these three families to control alpha (i.e. compared the lowest *p*-value with 0.05/3 = 0.0167 for the families with three variables and the lowest *p*-value with 0.05/2 = 0.025 for the families with two variables). Because of the non-parametric analysis of results, and the comparison of medians, effect sizes were calculated using *r*-values (Cohen, 1988).

Results for the first research question (effect of medication on *microstructure* language production elements during narrative production) were not significant (all *p*-values in the table were not less than the level of significance of 5%).

The second research question asked was whether there was an effect of medication on *macrostructure* language production elements (introduction, character development, conflict resolution, mental state, referencing, cohesion, coherence and total) during narrative production as measured by NSS. The *p*-value of 0.008, which is less than 0.05, indicates that MPH-OROS^®^ elicited a statistically significant effect on the overall narrative performance (NSS total score). Using the Holm procedure for multiple comparisons, results showed that the medication has a significant influence on one of the seven independent *macrostructure* language production elements: cohesion. The *p*-value for cohesion was 0.020, indicating that MPH-OROS^®^ elicited a significant effect on this component. Note that the improvement for conflict resolution failed to reach significance after the family-wise correction for multiple comparisons, *p* = 0.026; however, the effect size for this difference was -0.454, indicating a medium-to-large correlation (Cohen, 1988). The remaining five story grammar variables independently showed that the medication has no significant influence on them.

## Ethical consideration

Prior to the onset of data gathering, a detailed outline of the study and data gathering procedures, including the administration of medication, was presented to the appropriate committees for ethical consideration. Approval by the Department of Speech-Language Pathology and Audiology Research Committee was obtained, following which ethical clearance was given by the Faculty of Humanities Research Proposal and Ethics Committee at the University of Pretoria.

## Discussion

The purpose of this study was to investigate the effect of MPH-OROS^®^ on microstructure and macrostructure elements of language in children with ADHD and concomitant language impairment. Overall, independent results indicate that MPH-OROS^®^ positively impacts aspects of language macrostructure, namely cohesion and the overall narrative, although little effect (not significant) was noted in microstructure elements. Details regarding each of the questions will be discussed below.

### Microstructure

The first research question addressed the effect of medication on microstructure language production elements during story narration as measured by productivity, grammatical complexity and lexical diversity. Results indicated that MPH-OROS^®^ did not impact microstructure elements. These findings are in line with the results obtained by Francis et al. (2001) but contrary to Derefinko et al. (2009). These results may be attributed to the notions that: (1) the group participants included those with various ADHD subtypes and (2) linguistic impairments persist despite improved attention during a circumscribed narrative production task.

In the current study, measures of productivity did not improve as a result of MPH-OROS^®^; however, Derefinko et al. (2009) showed that stimulant medication increased productivity (number of clauses produced). Based on Tannock’s (2004/2005) summary of Fine’s (2005) ‘linguistic manifestation of ADHD symptoms’, excessive language output can be attributed to the hyperactive component of ADHD. Therefore, children’s specific ADHD presentations, namely the presence or absence of the hyperactive component, might impact the language production of children, particularly with regard to productivity.

Research has shown that performance on language tasks differs between individuals from different ADHD subtypes (Engelhardt et al., 2011; Engelhardt, Veld & Nigg, 2012). One notable feature of the performance in the current sample was the wide variability among individuals; however, we lacked the statistical power to differentiate between ADHD subtypes. Future research will be necessary to establish whether this variability might be accounted for by ADHD subtype and whether effects of medication differ across ADHD subtypes for the measures examined. It is hypothesised that medication may impact narrative microstructure differently for those with ADHD-PI and ADHD-C, such that improved attention may slow down narrative performance and increase productivity in former case and may result in more focused and therefore shorter narratives in the latter case.

Grammatical complexity and lexical diversity did not improve with the administration of MPH-OROS^®^. These results could provide support for the idea that, despite improved attention, linguistic impairment persists. Camarata and Gibson’s (1999) discussion of language acquisition in children with ADHD is congruent with this line of thinking. Through their review of ADHD and its impact on pragmatic skills, based on the transactional model of mother–child interactions (Sameroff & Chandler, 1975; Yoder & Warren, 1993), they suggest that it is this aspect of language that is particularly vulnerable to disruption because of inattentive, hyperactive and impulsive behavioural characteristics. They attribute linguistic deficits to pragmatic difficulties during early interactions between mother and infant. These negatively influence the child’s ability to engage in language-learning opportunities and may lead to cascading impairments in language ability in childhood and adolescence. Even with improved attention because of the administration of MPH-OROS^®^, children with ADHD may not have the requisite language skills to produce a more mature narrative.

### Macrostructure

The second research question addressed the effect of medication on macrostructure language production elements during story narration as measured by NSS. An overall improvement in NSS total score was observed following MPH-OROS^®^. Various categories of the NSS were differentially affected. One specific macrostructure element was improved (i.e. cohesion) while five were not (i.e. introduction, character development, mental states, referencing and conclusion). The improvement noted in cohesion may be indicative of the effect of MPH-OROS^®^ on executive functions as this element may be more deeply rooted in one’s present ability to relate and organise events in relation to one another and perhaps less reliant on the modelling of story structure during previous language-learning opportunities.

As predicted, MPH-OROS^®^ showed a positive effect on the cohesion of narratives, supporting the theory that the language characteristics of individuals with ADHD can be attributed to executive dysfunction (Tannock & Schachar, 1996). The improvements may be indicative of the documented effect of stimulant medications on some tasks of executive functions (Aman, Roberts & Pennington, 1998), thus allowing participants to better plan and organise their narratives, improved sequencing and smoother transitions between story events. Although conflict resolution did not significantly improve, it is an aspect of narrative ability that is worth future investigation as evidenced by the effect size. Although speculative, one possibility for the potential improvement in conflict resolution may be that MPH-OROS^®^ decreases impulsive behaviour, slowing the thought process and allowing one to tap into executive functions. Participants would therefore be able to select information, monitor the outcome of story events and redirect responses where necessary.

The results of the current study indicated improvement in aspects of narrative ability that were not identified by Derefinko et al. (2009), which may be due to differences in the method of elicitation. Derefinko et al. (2009) made use of online story narration, whereas the current study provided participants with the opportunity to preview the storybook prior to narrative production. As a result, participants were given the chance to plan and organise their story components in relation to one another, as well as to tell the story with the end goal in mind. The elicitation protocol in the current study may have increased the chance that participants, given their increased focus and attention because of the administration of MPH-OROS^®^, would include causal chains and make comments regarding goal-directed behaviour and initiating events.

In addition to cohesion, the administration of MPH-OROS^®^ significantly impacted the NSS total score, indicating meaningful improvement in the overall impression and efficacy of a narrative. Therefore, results suggest that some children with ADHD, who are treated with MPH-OROS^®^, may be better able to express their thoughts and experiences through more effective, richer narratives. The fact that mental states did not improve in this study is contradictory to the results obtained by Francis et al. (2001). In that study, MPH improved the recognition and verbal expression of internal responses. Internal responses refer to the emotional responses, thoughts and desires of characters (referred to in the current study as mental states). Francis et al. (2001) attributed this improvement to an increase in sensitivity to emotional information and the actions of others.

The fact that MPH-OROS^®^ did not improve performance on all aspects of narrative production is consistent with previous work focusing on the effects of stimulant medication on higher-order skills in children with ADHD, both on language and non-language tasks. Bailey, Derefinko, Milich, Lorch and Metze (2011) investigated the effect of MPH on free recall of story events in children with ADHD. Results indicated that although stimulant medication improved the percentage of story events that were recalled, there was no improvement in the recall of events that were central to the story. Abikoff et al. (2009) investigated the effect of stimulant medication on organisational skills, planning and time management and found that although aspects improved, medication did not eliminate difficulties altogether. Similarly, Pelham et al. (1990b) found that although stimulant medication improved immediate attention during baseball games, it did not improve their overall performance. These studies, along with the results for those of Derefinko et al. (2009), Francis et al. (2001) and the current study, suggest that although stimulant medication improves attention and concentration, it cannot make up for the loss of structural and pragmatic language abilities that may be associated with – or downstream effects of – the primary symptoms of ADHD. Therefore, although stimulant medication improves performance of individuals with ADHD in some domains, it is not a sufficient intervention to improve higher-order metacognitive and linguistic skills. A combination of treatments is therefore necessary to ensure that individuals with ADHD are able to reach their full potential. These interventions could include, but may not be limited to, medication, behaviour modification therapy, speech-language therapy, occupational therapy and remedial education.

### Limitations

The strict selection criteria and resultant small sample size of the current study did not allow for the differentiation between presentation subtypes of ADHD (i.e. ADHD-PI vs. ADHD-PH vs. ADHD-C). Differentiation between ADHD subtypes would prove valuable as the effect of MPH-OROS^®^ on narrative ability may be influenced by the presence or absence of the hyperactive component.

In addition, obtaining multiple narrative samples per participant would allow for a more accurate representation of an individual’s abilities as results would be less affected by external and internal factors. However, because of the nature of this study, it was necessary that material be similar with regard to theme, structural complexity, number of main characters and length so as to not exert an influence on the narratives produced. With limited material available to meet these criteria, it was only possible to obtain a single on- and off-medication narrative sample. Further, the repeated elicitation of narratives from the participants may have resulted in training effects and could be circumvented in future studies by making use of a crossover design or adjusting the period the period of time between on- and off-medication sampling.

Developmental differences in narrative production, because of the wide age range of the study participants, may have impacted upon their narrative ability and could have subsequently influenced the results.

Although reliability appeared low for the introduction component on the NSS, it is important to note that raw scores never differed by more than a single point (e.g. 2 [intermediate between immature and emergent] vs. 3 [emergent]). Scores within one point are considered consistent, given that the scale was constructed with clear anchors of 1, 3 and 5 (with intermediate scores of 2 and 4). In addition, the low scores can in part be attributed to the very small number of reliability transcripts. Rescoring 20% of the data translated into only five language samples, leaving little margin for error. With regard to the difference in points between scorers, no trends could be noted between transcriptions with regard to participant characteristics for the two transcripts with a scoring discrepancy. Transcripts were both 100% intelligible, and the book used to elicit the narratives differed between transcripts as did the presence or absence of medication. Reliability for conflict resolution was also low; however, again, scores never differed by more than a single point and scores were identical for four of five transcripts.

One possible reason for differences between scorers may be based on the scorers’ interpretation of the descriptions of the criteria. For example, an emergent score (3 points) under the introduction category requires (1) Setting: ‘states general setting but provides no detail’, ‘descriptions or elements of setting are given intermittently through story’ and ‘may provide description of specific element of setting (e.g. the frog is in the jar)’ and (2) Characters: ‘characters of story are mentioned with no detail/description’. The immature (1 point) requires that the speaker ‘Launches into the story with no attempt to provide setting’ (Miller et al., 2011). It is therefore clear whether or not to assign a score of 1, but far more challenging to determine whether the performance meets all the descriptors to warrant a score of 3. For example, the interpretation of descriptions given ‘intermittently’ throughout the story may differ between scorers. Should a scorer’s best judgment be that not all descriptions were met; a score of 2 should be awarded instead. Similar discrepancies were reported in Petersen et al.’s (2008) summary and evaluation of measures of narration, which included the NSS, noting particular discrepancies with the coding of mental states and cohesion (Petersen et al., 2008).

### Future directions

Although the current study focused on the effect of medication on the narrative ability of children with ADHD using a within group comparison, future research should be carried using a peer control group comprising typically developing children. This would provide further clarity regarding the narrative difficulties experienced by this population as well as insights into the degree to which medication normalises the narrative performance of children with ADHD.

The current study should be replicated using a larger sample size. This would allow for the differentiation between ADHD subtypes, namely ADHD-PI, ADHD-PH and ADHD-C, as recent studies have noted differences in language production between these groups (Engelhardt et al., 2011, 2012), and would make generalisation of the findings possible. This may provide further insights into the relationship between ADHD and language impairment as well as the effect of MPH-OROS^®^ on language production. Further studies should also be carried out to determine whether the effects of stimulant medication on narrative ability are mediated by attention or memory by directly assessing attention, memory and executive function.

Future studies in this line of research would benefit from the comparison of language samples elicited in different language sampling contexts (e.g. conversation, expository samples and persuasion), which may be maximally informative in terms of the effects of stimulant medication on the spontaneous expressive language of children with ADHD.

To support research in this area, additional elicitation materials should be developed to allow multiple narrative samples – or multiple samples from other contexts – to be obtained over time from a wider age range of participants. For example, a series of standardised narrative elicitation materials would allow for repeated measurements without the reliability of narrative production being affected by additional factors such as story length, number of characters and subject matter. Although there are six wordless picture books available by Mercer Meyer, the literature suggests that these books may not all be sufficiently comparable for the elicitation of multiple narratives over time. John et al. (2003) examined children’s retelling of narratives using the Strong Narrative Assessment Procedure (SNAP) (Strong, 1998). The SNAP contains the wordless picture books *Frog, Where Are You?, A Boy, a Dog and a Frog* (Mayer, 1967) and *One Frog Too Many*, as well as *Frog Goes to Dinner*, which is included as practice material. Through the exploration of the equivalency of the stories, John et al. found that *A Boy, a Dog and a Frog* was retold with greater ease, resulting in inflated scores for story grammar components and inferential comprehension when compared with other two stories. Based on these results, it was recommended that clinicians only administer *Frog, Where Are You?* or *One Frog Too Many* – the two books utilised in the present study – when assessing progress in narrative production. As a result, one’s ability to obtain multiple samples over time is limited.

If researchers are able to limit the age range of participants more narrowly, some available tools may prove beneficial in furthering our understanding of the effect of stimulant medication on narratives in children with ADHD. This includes the Multilingual Assessment Instrument for Narratives (MAIN) (Gagarina et al., 2012), which can be used for children aged 3–10. Normed protocols that may prove useful include the Test for Narrative Language (TNL) (Gillam & Pearson, 2004), although this only contains two story sequences and will therefore not allow for multiple data gathering sessions over time, and the Edmonton Narrative Norms Instrument (ENNI) (Schneider, Dubé & Hayward, 2005), which can be used with participants aged 4–9.

### Clinical significance

The findings of this study have potential implications, both academically and socially. Children with ADHD are at risk for academic underachievement because of the primary symptoms associated with ADHD (Loe & Feldman, 2007). In addition, the documented narrative impairment may further hamper academic success in this population (Moonsamy et al., 2009). Narrative ability is critical to classroom performance and forms part of common daily activities (Kaderavek & Sulzby, 2000). Individuals with poor narrative ability are often negatively perceived by others (Hemphill & Siperstein, 1990). Furthermore, narrative ability fosters social communication, allowing one to engage with their peers (Coupland & Jaworski, 2003), thus supporting socio-emotional development.

Therefore, because of the large role that narratives play in academic and social settings, it is evident that narrative inability in children with ADHD cannot be ignored by those professionals working with this population. The findings that MPH-OROS^®^ improves aspects of narrative ability in children with ADHD could be valuable information for clinicians and speech-language pathologists as this, if replicated, might prove beneficial when guiding parents in decision-making processes regarding medication as well as the ongoing necessity of speech-language therapy in children with ADHD.
